# Comprehensive Care for Patients with Sarcoidosis

**DOI:** 10.3390/jcm9020390

**Published:** 2020-02-01

**Authors:** Catharina C. Moor, Vivienne Kahlmann, Daniel A. Culver, Marlies S. Wijsenbeek

**Affiliations:** 1 Department of Respiratory Medicine, Erasmus Medical Center Rotterdam, 3015 GD Rotterdam, The Netherlands; 2 Department of Pulmonary Medicine, Respiratory Institute, Cleveland Clinic, Cleveland, OH 44195, USA

**Keywords:** sarcoidosis, quality of life, comprehensive care, holistic management

## Abstract

Sarcoidosis is a multisystem granulomatous disease, associated with significant morbidity and impaired quality of life. Treatment is aimed at recovering organ function, reducing symptom burden and improving quality of life. Because of the heterogeneity and variable disease course, a comprehensive, multidisciplinary approach to care is needed. Comprehensive care includes not only pharmacological interventions, but also supportive measures aimed at relieving symptoms and improving quality of life. The purpose of this review is to summarize the most recent knowledge regarding different aspects of care and propose a structured approach to sarcoidosis management.

## 1. Introduction

Sarcoidosis is a multisystem granulomatous disease of unknown cause, that can affect almost any organ. The past decade we have gained more insights in the dysregulation of the immune system, which is thought to play an important role in the etiology of sarcoidosis [1]. Patients may present with a wide range of organ-specific symptoms, such as cough and dyspnea, or non-organ manifestations including fatigue, depression, and reduced exercise tolerance [1]. In about 60 percent of patients, remission occurs spontaneously or after treatment within 10 years after diagnosis [2]. In approximately 10–40% of patients, sarcoidosis becomes chronic and progressive. Mortality is around 1–5%, and is higher in African-American patients and elderly patients [3]. In general, sarcoidosis leads to a substantial economic burden and societal impact, mainly because of hospitalizations, medication costs and the inability to work [3,4]. For individual patients, high symptom burden often leads to psychological problems and an impaired quality of life (QoL) [5].

**Figure 1:**
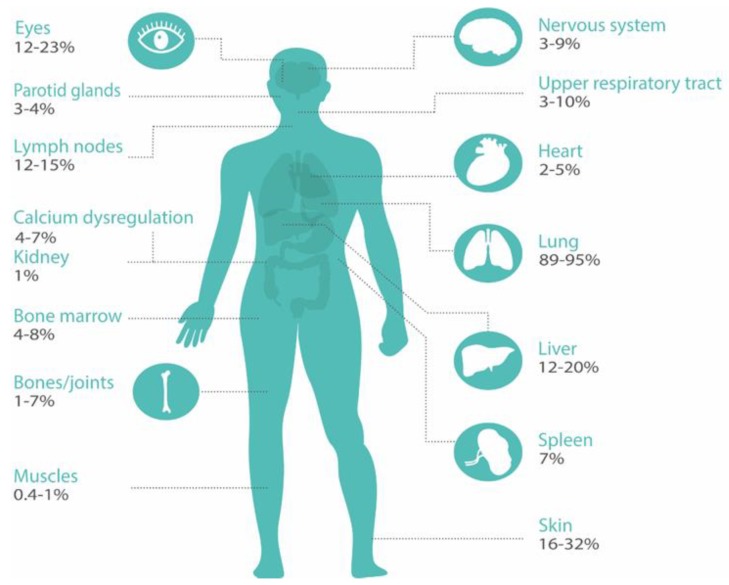
Organ involvement in sarcoidosis. Organ involvement is classified according to the ACCESS organ assessment instrument [8]. Prevalence data are used from references [1,9].

Current pharmacological treatment of sarcoidosis is usually immunosuppressive and directed at decreasing granulomatous inflammation [2]. Overall, treatment is aimed at recovering organ function, reducing symptom burden and improving quality of life [6]. Pharmacological interventions are not curative and—in a subgroup of patients—symptoms or disease progression may persist [2]. A comprehensive approach to care is needed for patients with sarcoidosis, especially because of the heterogeneity in symptoms and organ involvement and variable disease course (Figure 1) [7]. This review is written from a pulmonologist’s perspective, as in many hospitals the pulmonologist is the central care coordinator. However, we would like to stress the importance of multidisciplinary care as extrapulmonary disease is present in the majority of patients.

The aim of this review is to summarize the most recent knowledge regarding different aspects of care in sarcoidosis and propose the “ABCDE model for sarcoidosis”, which is an adaptation of the version in interstitial lung diseases (ILD) [10,11].

## 2. ABCDE Model

**Figure 2:**
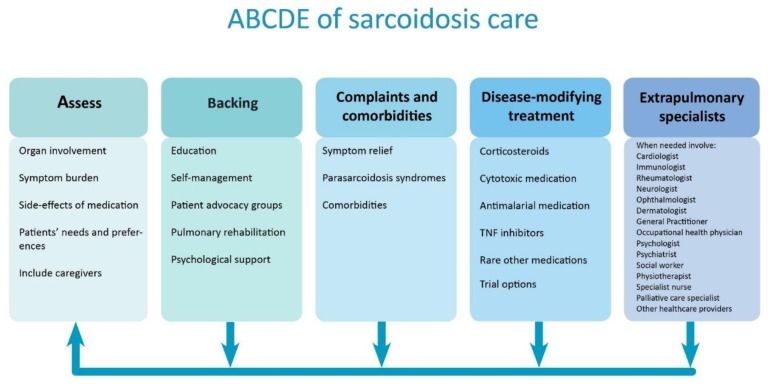
Structured approach to comprehensive care in sarcoidosis. The arrows show that individual patient’s symptoms, needs, and treatment should regularly be reassessed. TNF = tumor necrosis factor.

The importance of comprehensive care in sarcoidosis is generally acknowledged [12,13]. Here, we describe the ABCDE model, that can be used to structure comprehensive sarcoidosis management in order to improve QoL and outcomes for patients (Figure 2). This model includes the following components: the Assessment of symptoms and patient’s needs, Backing patients by providing support and education, treatment of Complaints and Comorbidities, Disease-modifying treatment, and the involvement of Extrapulmonary specialists. As disease activity, organ involvement, and patients’ preferences may vary during the disease course, regular reassessment is essential. The ABCDE model can provide guidance to clinicians during the first work-up and follow-up of patients with sarcoidosis. Different components of the model are discussed in more detail in the following paragraphs. 

### 2.1. Assess

In all sarcoidosis patients, organ involvement should be carefully assessed in the diagnostic process and during follow-up. The degree of organ damage and disease activity is often difficult to quantify, as no gold standards exist and symptoms are often non-specific [3,14,15]. Reported percentages of organ involvement are wide ranging, depending on whether only clinically overt organ involvement or also asymptomatic organ involvement is taken into account. For example, 2%–5% of patients have symptomatic cardiac involvement, but the frequency of clinically inapparent involvement is much higher (probably up to 25%) [9]. 

Furthermore, organ involvement significantly varies for different ethnic groups. In individual patients, the number of involved organs may change over time and therefore diagnostic assessments have to be performed regularly, particularly if patients express new symptoms [1,16]. The frequency in which different organ systems are affected in sarcoidosis is summarized in Figure 1 [1,9]. Consequently, patients without apparent organ involvement could still have a high symptom burden [13]. Hence, Drent et al. propose four different domains that should be evaluated in the complete work-up of a patient; not only the severity, extent and activity of the disease, but also the impact of disease [13]. In a recent survey with over 1000 respondents, 95% of patients reported sarcoidosis-related symptoms, and self-reported symptom burden of sarcoidosis was high [17]. Due to this high symptom burden, uncertain prospects, and sometimes social isolation and inability to work, sarcoidosis has a huge impact on lives of patients [3,13,18]. In a Dutch government survey, more than 60% of sarcoidosis patients (*n* = 150) considered their general health moderately to severely impaired. Only 7% answered that their health problems had no influence on their social life [19]. Furthermore, almost 50% of patients in a recent study (*n* = 755) were partially or totally unable to work due to their sarcoidosis, highlighting the considerable impact of sarcoidosis on daily life [18]. A Swedish national registry evaluation of 3347 sarcoidosis patients aged 25–59 years, suggested 8% lower income and 26 lost work days in the year of diagnosis compared with age matched controls [20]. In a US registry (*n* = 2318), 44% of respondents reported a large effect on household finances, and 31% had to quit their job after the diagnosis of sarcoidosis [4].

Overall, sarcoidosis patients have an impaired QoL compared with the healthy population [5]. Several studies analyzed the relation between symptoms and QoL in sarcoidosis. Symptoms predictive for QoL are depression, anxiety, fatigue, reduced exercise capacity, SFN-related symptoms, dyspnea, pain, and arthralgia [15,21,22,23,24,25,26]. Interestingly, partners of sarcoidosis patients also experience a reduced QoL compared with healthy controls [21]. Moreover, partners tend to have increased anxiety levels and psychological distress [27]. Both patients as well as their partners reported that there should be more support for partners of sarcoidosis patients [27].

QoL can be defined as “an individual’s perception of their position in life” and is influenced by a person’s values, beliefs, culture, physical health, social and psychological state [28]. Qol can be measured with patient-reported outcome measures (PROMs). PROMs are instruments that “collect self-reported information about a patients’ health condition, without any intervention from a healthcare provider” [29]. PROMs can be either generic (applicable to the whole population), disease-specific (developed or validated in a specific disease) or domain-specific (assessing severity or burden of a specific symptom or organ). A wide range of PROMs are currently being used for sarcoidosis [30,31]. A number of these instruments, such as the King’s Sarcoidosis Questionnaire (KSQ), Sarcoidosis Health Questionnaire (SHQ), Sarcoidosis Assessment Tool (SAT), and Fatigue Assessment Scale (FAS) have been specifically developed to measure QoL and symptom burden in sarcoidosis [31,32,33,34]. Although PROMs are mainly used in clinical trials, well-validated PROMs could also be used in clinical practice to evaluate treatment effect and longitudinal changes in symptoms and QoL [31,35]. In other chronic diseases, the use of PROMs in regular care is associated with enhanced communication and shared-decision making, detection of unrecognized problems, higher patient satisfaction and improved QoL [36,37]. Future research could affirm whether this is also the case in sarcoidosis.

Other factors with a potential negative impact on QoL should not be forgotten in the assessment of sarcoidosis patients. For example, medication for sarcoidosis may lead to debilitating side-effects, such as weight gain, diabetes, osteoporosis and psychological problems [38,39,40]. According to one study, patients with higher cumulative doses of prednisolone had a significantly lower QoL when adjusted for disease severity [4,39]. Moreover, medication-related events lead to a substantial number of hospitalizations in patients with sarcoidosis [41]. Consequently, (dis)advantages of starting and continuing therapy should be weighted by the healthcare provider and patient during every clinic visit. 

Although it is increasingly acknowledged that patient perspectives are important for optimizing individually-tailored treatments [17,27,42], literature concerning (unmet) needs and preferences of patients with sarcoidosis is scarce. Recently, an international survey revealed that sarcoidosis patients considered QoL and functionality as the most important treatment outcomes [7]. Blood tests and pulmonary function tests were considered the least important outcomes [7]. These results are in contrast to the current focus of most clinicians on physiological outcome measures [5,15,17,30]. In a number of studies, patients reported the need for better information about sarcoidosis and shared decision making [19,27]. As treatment goals can obviously differ between patients, the first step in shared-decision making is identifying patients’ needs and preferences. During the disease course, patients should be involved in their treatment plan and in the regular evaluation of benefits versus risks of (pharmacological) treatment [12,16,19]. Including patients as a partner in care could lead to better QoL and adherence to treatment [43,44]. Multidisciplinary management and improved access to sarcoidosis specialists and expert centers for sarcoidosis are other important needs for patients, although it must be acknowledged that not all sarcoidosis patients require tertiary care [7,27].

### 2.2. Backing

Several support measures to improve QoL for sarcoidosis patients have been suggested in the past years. Better information and education are vital to optimize care for sarcoidosis [13,18,27,45,46]. Even in the current internet era, patients state that they cannot find enough reliable information about their disease online [19,27] Moreover, the complexity and heterogeneity of sarcoidosis may complicate communication and knowledge transfer between healthcare providers and patients [13]. Currently, patients and their partners often feel misunderstood because of a lack of knowledge among healthcare providers and the general public [13,27]. Hence, awareness should be raised in society and among relevant healthcare providers. Patient advocacy groups could play an important role in providing understandable information and education, by organizing information meetings and awareness campaigns [47]. Although support groups can have obvious benefits, effects on QoL have never been studied in sarcoidosis.

Self-management support is one of the main pillars of the chronic care model, developed to improve care for patients with chronic conditions [48]. Many aspects of chronic disease care can only be managed by patients themselves. Self-management strategies cover all disease domains, and include for example behavioral changes, medication use, exercise, dietary strategies and home monitoring of disease [49,50]. To achieve skills for self-management, patients and families need to be adequately trained and supported by their healthcare providers [1]. Use of novel eHealth solutions to enhance self-management has recently gained interest in sarcoidosis [45,51]. Self-monitoring of symptoms, side-effects, QoL, activity and pulmonary function at home has shown to be feasible and highly appreciated by patients with sarcoidosis [51,52]. A comprehensive home monitoring program may provide patients with more insights in their disease course, and thereby empower patients and enhance communication with healthcare providers. In a recent study, home monitoring of pulmonary function allowed for earlier detection of steroid treatment effects, suggesting that patient-managed steroid dosing regimens may be feasible [52].

While activity tracking at home could possibly help to stimulate exercise [51,53,54,55], supervised training programs or multidisciplinary pulmonary rehabilitation might have more beneficial effects [13]. Current evidence indicates that a structured, supervised exercise program can improve symptoms, QoL, exercise capacity, and muscle strength in patients with different stages of sarcoidosis [56,57,58,59,60,61]. Although these studies showed promising results, no evidence-based guidelines exist to date. In a survey of international sarcoidosis experts, the vast majority of respondents considered physical training in sarcoidosis valuable and would recommend it as standard of care [56]. Pulmonary involvement, fatigue, and reduced exercise tolerance are the main indications for physical therapy in sarcoidosis [56]. The long-term effects of physical therapy, optimal duration and content of exercise programs in sarcoidosis have never been investigated and need further study. Furthermore, reimbursement and distance to appropriate exercise programs vary between regions and countries and may limit access to physical therapy for sarcoidosis patients [56]. Telerehabilitation could potentially be a solution for patients living in rural areas because distances are bridged online. A study evaluating the feasibility and effect of a telerehabilitation program in sarcoidosis is ongoing (NCT03914027).

The majority of patients would like to have better access to psychological support [7,27]. In clinical practice, patients are referred to a psychologist or psychiatrist for further counseling and treatment on indication, though some people advocate for standard psychological assessment [62]. Cognitive behavioral therapy has been proposed as a promising method to offer psychological support; this therapy could potentially improve patients’ coping strategies and thereby reduce stress, anxiety and depression [13,23]. To date, one study analyzed the impact of mindfulness-based exercise therapy on physical and psychological symptoms in sarcoidosis. Even though this was a modified training consisting of only one 45-minute workshop, symptoms significantly decreased after the session [63]. A randomized controlled trial to assess the impact of an online mindfulness-based cognitive behavioral therapy on QoL, fatigue, stress, and anxiety is currently ongoing [64]. 

### 2.3. Complaints and Comorbidities

Symptom relief is a major aspect of sarcoidosis management. Dyspnea is among the most common symptoms in sarcoidosis and is an important indication for treatment [12]. Dyspnea is often multifactorial and can be caused by pulmonary, musculoskeletal or cardiac involvement of sarcoidosis, or deconditioning. Other causes for dyspnea such as infection or pulmonary hypertension should also be evaluated [12]. Non-pharmacological treatment options include physical training, multidisciplinary pulmonary rehabilitation and potentially cognitive behavioral therapy [58,59,60,63] (Table 1). Cough is present in up to 53% of sarcoidosis patients [65]. Patients with sarcoidosis have a significantly higher cough frequency compared with the normal population [66]. Even though cough is often part of the disease, other causes such as reflux or post-nasal drip should always be excluded. One study showed that inhaled corticosteroids may be effective in reducing cough in sarcoidosis, but two other small studies showed no effects of inhaled corticosteroids on cough in sarcoidosis [67,68,69]. Consequently, inhaled corticosteroids should not be routinely administered unless a trial demonstrates efficacy [70]. In the recent CHEST guideline, speech therapy is recommended for patients with ILD and refractory cough, however, this therapy has not been specifically evaluated in sarcoidosis [70]. Vasoactive intestinal peptide (VIP) inhalation seemed to reduce cough in sarcoidosis in one small open clinical phase II study, but has never been investigated in a randomized setting [71]. Further studies regarding antitussive therapy in sarcoidosis are highly needed.

Non-organ manifestations or parasarcoidosis syndromes include symptoms as fatigue, depression, anxiety, pain, SFN, and cognitive impairment [46]. As most of these symptoms are related to each other, it can be challenging to avoid a vicious circle. Fatigue is one of the most prevalent (up to 90% of patients) and burdensome symptoms for patients with sarcoidosis [17,27]. Fatigue is a complex, multifactorial problem [13]. Symptoms as sleep disturbance, psychological problems, cognitive impairment, reduced exercise capacity, and muscle strength are all linked to fatigue [77,78,79,80,81]. Moreover, comorbidities and medication use may also contribute to fatigue. Previously, it has been shown that patients with multi-organ involvement and a higher number of comorbidities have increased fatigue levels [78]. Comorbidities associated with fatigue are sleep apnea, pulmonary hypertension, diabetes mellitus, thyroid disorders, and obesity [78]. Fatigue is often a chronic problem, which persists or worsens despite sarcoidosis treatment. Research into better pharmacological and non-pharmacological treatment options for sarcoidosis-associated fatigue is scarce. Two small randomized trials demonstrated that neurostimulants (armodafinil and methylphenidate) have the potential to reduce sarcoidosis-associated fatigue [72,73]. A number of other relatively small or retrospective studies showed the benefits of physical training and cognitive behavioral therapy on fatigue in sarcoidosis [57,58,59,60,63]. Larger studies investigating the etiology and better treatment options for sarcoidosis-associated fatigue are essential to improve symptom burden and QoL for patients. 

Next to fatigue, SFN-related related symptoms were reported by the vast majority of patients (86%) in a recent European survey [17]. SFN is difficult to diagnose and to treat [13]. Patients may experience a myriad of symptoms, but frequently present with neuropathic pain or autonomic dysfunction [76]. SFN is usually treated with anticonvulsants, antidepressants, topical anesthestics or opioids [46]. However, these standard treatment options are often not effective [13]. Retrospective studies showed that intravenous immunoglobulin (IVIG) and tumor necrosis factor (TNF) inhibitors might be effective in reducing SFN-related symptoms [75,76]. Furthermore, the erythropoietin agonist cibenitide (ARA290) showed promising results on corneal nerve fiber abundance in a phase 2b clinical trial [82]. No statistical differences were found in patient-reported outcomes, probably due to the design of the study [82].

Other common non-organ manifestations of sarcoidosis are psychological problems. Previous studies reported anxiety in up to 33% of patients and depressive symptoms in up to 66% of patients [46,83]. In a study which used a comprehensive diagnostic interview, the prevalence of major depressive disorder remained strikingly high, at 25% [62]. Most other studies used questionnaires to screen for depressive symptoms [5,84,85]. Patients with depression or anxiety tend to have a higher symptom burden and different perception of disease severity [83]. Pulmonary rehabilitation may have a beneficial effect on psychological wellbeing [58]. Cognitive behavioral therapy also has the potential to improve stress, anxiety and depression in sarcoidosis [63]. Furthermore, regular pharmacological therapy for depression and anxiety (antidepressants and anxiolytics) could be offered after an appropriate psychiatric evaluation [63].

Cognitive problems, including memory loss, concentration difficulties and impaired short-term memory are reported by more than 50% of sarcoidosis patients [17,81]. At present, the cause of cognitive failure is considered multi-factorial, and possibly related to chronic inflammation. Currently, no convincing therapies or interventions are available. An observational study indicated that TNF inhibitor treatment may have a beneficial effect on cognition, as measured by the cognitive failure questionnaire (CFQ) [74]. Besides, treatment of associated symptoms may also ameliorate cognitive function.

Comorbidities may also importantly impact QoL and are more prevalent in patients with sarcoidosis compared to the normal population [41,86,87]. A higher number of comorbidities is associated with more frequent hospitalizations and a higher mortality rate [87,88]. The development of new comorbidities, related either to steroid use or to sarcoidosis itself, after the diagnosis of sarcoidosis is independently and strongly associated with a number of adverse outcomes, including worse QoL, risk of hospitalization, and financial impacts [4]. One study showed that more than half of sarcoidosis patients have more than one comorbidity, with arterial hypertension, thyroid disorders and diabetes mellitus being the most prevalent comorbidities [86]. Another study found a significant higher prevalence of chronic liver disease, autoimmune diseases, chronic pulmonary diseases and cancer in patients with sarcoidosis [87]. A study in African-American sarcoidosis patients reported that 90% of patients had one or more comorbidities [89]. The number of comorbidities is higher in older patients, multi-organ involvement and patients with lower incomes [4,86]. Patients with comorbidities obviously have a higher disease complexity, making multidisciplinary management of sarcoidosis even more essential [87]. The presence of comorbidities should therefore be carefully (re)assessed during the disease course.

### 2.4. Disease Modifying-Treatment

Not all patients with sarcoidosis require pharmacological treatment, as the majority will have spontaneous regression of the disease [2]. Treatment is primarily aimed at suppression of the immune system, and thereby preventing organ damage. The main reasons to start treatment are “to avoid danger or improve quality of life” [90]. Factors which should be taken into account are the probability of spontaneous resolution, risk for disease progression, extent of disease, organ dysfunction, activity of sarcoidosis, symptom burden and patient’ preferences [2,5,12,16].

Pharmacological treatment of pulmonary sarcoidosis should be considered for patients with significant pulmonary symptoms and patients with an impaired or deteriorating lung function [16,91]. For extrapulmonary sarcoidosis, the ATS/ERS/WASOG guidelines state that treatment should always be initiated in case of cardiac sarcoidosis, involvement of the central nervous system, hypercalcemia and ocular sarcoidosis not responding to topical treatment [92]. Other common indications for treatment are hepatic involvement (impaired liver function, portal hypertension), splenic involvement, bone marrow involvement (cytopenia), nephrolithiasis and skin involvement with disfiguring lesions [16]. Treatment may be initiated in patients who have disabling symptoms without organ damage; however, this should always be a shared-decision with patients as medication can have debilitating side-effects [12,16]. Consequently, efficacy and side-effects of treatment should be assessed during every outpatient clinic visit. 

The current guideline, dating from 1999, states that “the appropriate treatment has not been well-defined for all patients” [92]. Presently, oral corticosteroids (e.g., prednisolone) are recommended as the first-choice therapy for sarcoidosis [92]. This recommendation is mainly based on expert opinion and a few relatively small observational studies and low-quality randomized trials from over 20 years ago [91,93]. Older studies have demonstrated that corticosteroids lead to an improvement in lung function, especially in patients with initial severe impairment of lung function [94,95]. Although corticosteroid treatment leads to short-term improvement of lung function, radiological improvement, and symptom reduction, previous studies have not conclusively demonstrated a beneficial effect in preventing disease progression [16,91]. Disease relapse occurs in over 30% of patients after discontinuation of corticosteroids. Furthermore, due to the lack of larger randomized trials the optimal dosage and duration of treatment remains unclear [93]. 

The most frequently used second-line treatment is the folic acid antagonist methotrexate [96]. Methotrexate has a significant steroid-sparing effect and improves lung function [97,98]. Methotrexate is increasingly used as first-line agent in case of (relative) contra-indications for corticosteroids [96]. A second choice second-line treatment is azathioprine. A retrospective study in the Netherlands and Belgium showed that azathioprine and methotrexate were equally effective, but azathioprine appeared to have more side-effects [97]. Mycophenolate mofetil and leflunomide are other second-line alternatives [1]. Antimalarial medication (chloroquine, hydroxychloroquine) is regularly prescribed in patients with cutaneous involvement or hypercalcemia [99,100]. 

In refractory sarcoidosis, TNF inhibitors can be prescribed as a third-line agent. Infliximab has been studied in randomized controlled trials and may have beneficial effects on both pulmonary and extra-pulmonary sarcoidosis in a subgroup of carefully selected patients [101,102,103]. Adalimumab also appears to be effective [104]. Other treatment options have recently emerged for patients with progressive sarcoidosis. The INBUILD study showed that nintedanib is effective in reducing forced vital capacity decline in patients with fibrotic interstitial lung disease, including sarcoidosis [105]. The efficacy of pirfenidone in progressive fibrotic sarcoidosis is also being studied (NCT03260556). Recently, inhibition of the JAK-STAT signaling pathway has been identified as a novel promising treatment target in sarcoidosis; prospective research is ongoing (NCT03910543, NCT03793439) [106]. Not only is more research needed for refractory sarcoidosis, but better evidence-based treatment for first-line therapy, aiming at a better balance between effects and side-effects, is also highly needed. A multicenter trial evaluating the efficacy and side-effects of prednisone and methotrexate as first-line treatment for pulmonary sarcoidosis has recently started in the Netherlands. A detailed description of medication for sarcoidosis is outside the scope of this review; for an extensive overview, we refer you to other published reviews specifically focusing on this topic [1,2,12,16,90,93].

### 2.5. Extra Pulmonary Specialists

Pulmonary physicians play an important role in the management of sarcoidosis patients, as the lungs are affected in up to 90% of patients. Nevertheless, a multidisciplinary team is needed to improve efficiency of care and outcomes for patients, as many organs can be affected and symptoms are wide-ranging [7,107]. While it is quite obvious that other medical specialists contribute their expertise in cases of extra pulmonary sarcoidosis, healthcare providers such as occupational health physicians, pain specialists or specialist nurses should not be forgotten [7,18] (Figure 2). Work participation is lower in patients with sarcoidosis; patients have more health-related sick days and a substantial income loss compared with the normal population [3,4,18,20]. Consequently, many patients undergo work capacity assessments and occupational health physicians need to be well-educated about sarcoidosis [13,18]. Although the role of specialist nurses is not as established as in other interstitial lung diseases, specialist nurses could function as coordinators of care in sarcoidosis and give patients practical and emotional support. In a small minority of patients, disease will progress despite all treatment lines. In a subgroup of these patients, lung transplantation may be an option. Furthermore, even though mortality is overall low in sarcoidosis, a multidisciplinary approach should also include palliative care specialists in end-stage disease or in case of a high disease burden.

## 3. Conclusions

A comprehensive, multidisciplinary approach is essential to treat patients with such a heterogeneous disease as sarcoidosis. Besides aiming at disease modification with pharmacological interventions, patients should also be offered supportive comprehensive care aimed at relieving symptoms and optimizing QoL. Patients’ preferences should be guiding all treatment decisions. To allow for better evidence-based treatment in the future, more research into both pharmacological and non-pharmacological treatment options is highly needed.

## Figures and Tables

**Table 1:** Overview of pharmacological and non-pharmacological treatment options for common sarcoidosis symptoms. Many of these recommendations are expert opinion or based on small studies.

Symptom	Pharmacological Treatment	Non-Pharmacological Treatment
**Dyspnea**	Regular disease-modifying treatment Treat other causes Supplemental oxygen (in case of hypoxemia)	Physical training, pulmonary rehabilitation [58,59,60] Cognitive behavioral therapy [63]
**Cough**	Regular disease-modifying treatment Treat other causes Inhaled corticosteroids *	Multimodality speech pathology therapy [70]
**Fatigue**	Treat other causes and comorbidities Neurostimulants: armodafinil, (dex)methylphenidate [72,73] TNF inhibitor treatment [74]	Treat reversible causes i.e., obstructive sleep apnea, obesity, depression Physical training or pulmonary rehabilitation [57,58,59,60] Psychosocial counselling [13] Cognitive behavioral therapy [63]
**Depression and Anxiety**	Antidepressants [13] ** Anxiolytics **	Cognitive behavioral therapy [63] Pulmonary rehabilitation [58] Psychological counselling [46]
**Small-Fiber Neuropathy**	Antidepressants Anticonvulsants Topical anesthetics Opioids [46] Intravenous immunoglobulin [75,76] TNF inhibitor treatment [76]	Mindfulness-based therapy [46]
**Cognitive Impairment**	TNF inhibitor treatment [74]	

* earlier studies showed conflicting results. ** should only be initiated after an appropriate psychiatric evaluation. TNF = tumor necrosis factor.
